# Lamin B2 Modulates Nucleolar Morphology, Dynamics, and Function

**DOI:** 10.1128/MCB.00274-17

**Published:** 2017-11-28

**Authors:** Ayantika Sen Gupta, Kundan Sengupta

**Affiliations:** Biology, Indian Institute of Science Education and Research, Pune, India

**Keywords:** lamin, nucleolin, nucleolus, nucleophosmin, nucleus, rDNA, rRNA

## Abstract

The nucleolus is required for ribosome biogenesis. Human cells have 2 or 3 nucleoli associated with nucleolar organizer region (NOR)-bearing chromosomes. An increase in number and altered nucleolar morphology define cancer cells. However, the mechanisms that modulate nucleolar morphology and function are unclear. Here we show that in addition to localizing at the nuclear envelope, lamin B2 localizes proximal to nucleolin at the granular component (GC) of the nucleolus and associates with the nucleolar proteins nucleolin and nucleophosmin. Lamin B2 knockdown severely disrupted the nucleolar morphology, which was rescued to intact and discrete nucleoli upon lamin B2 overexpression. Furthermore, two mutually exclusive lamin B2 deletion mutants, ΔHead and ΔSLS, rescued nuclear and nucleolar morphology defects, respectively, induced upon lamin B2 depletion, suggesting independent roles for lamin B2 at the nucleolus and nuclear envelope. Lamin B2 depletion increased nucleolin aggregation in the nucleoplasm, implicating lamin B2 in stabilizing nucleolin within the nucleolus. Lamin B2 knockdown upregulated nucleolus-specific 45S rRNA and upstream intergenic sequence (IGS) transcripts. The IGS transcripts colocalized with aggregates of nucleolin speckles, which were sustained in the nucleoplasm upon lamin B2 depletion. Taken together, these studies uncover a novel role for lamin B2 in modulating the morphology, dynamics, and function of the nucleolus.

## INTRODUCTION

The nucleolus is the largest nuclear subcompartment and is the site of ribosomal DNA (rDNA) transcription, processing, and ribosome biogenesis (1). The nucleolus undergoes cycles of disassembly and reassembly during mitosis (2). At the end of mitosis, small prenucleolar bodies (PNBs) assemble on human chromosomes 13, 14, 15, 21, and 22 bearing the nucleolar organizer regions (NOR), which then coalesce to form the nucleolus (3). Altered nucleolar numbers and structure correlate with cancers and ribosomopathies (4, 5).

Electron microscopy (EM) revealed that the nucleolus in amniotes has a tripartite organization consisting of the innermost fibrillar compartment (FC), the intermediate dense fibrillar compartment (DFC), and the outermost granular component (GC) (6). Inhibition of nucleolar transcription, rRNA processing, or assembly into preribosomes perturbs the integrity of the nucleolar compartments. Inhibition of rRNA transcription by actinomycin D (Act D) induces nucleolar cap formation, inversion of the FC, DFC, and GC, and dispersion of nucleolar proteins into the nucleoplasm, underscoring the role of active transcription of rDNA in maintaining nucleolar integrity (7). Thus, nucleolar morphologies are affected by altered metabolic rates and physiological stresses, such as nutrient deprivation, DNA damage, and hypoxia, that inhibit rDNA transcription (8–11). On the other hand, hyperproliferative cancer cells and hypertrophic cardiomyocytes with elevated protein synthesis and rDNA transcription show increased numbers of or enlarged nucleoli (12, 13). Often these nucleoli are irregular in morphology and therefore serve as prognostic markers of carcinogenesis (14). The nucleolar structure is also altered when rRNA processing is compromised upon depletion of the rRNA-processing factors NF90/NF110 or the key ribosomal proteins uL18 (RPL5) and uL5 (RPL11) (15, 16).

Mass spectrometric analysis of isolated nucleoli identified ∼4,500 nucleolar proteins that modulate ribosome biogenesis, cell cycle control, and DNA damage repair (17). However, the majority of nucleolar proteins remain uncharacterized in terms of their potential to modulate nucleolar structure and function. Upstream binding factor (UBF), nucleolin, and nucleophosmin are well-characterized nucleolar proteins that are essential for nucleolar formation and morphology (18–21). Fibrillarin and nucleophosmin, which are associated with ribosome biogenesis, phase-separate from the soluble nucleoplasm into nucleoli in an rRNA-dependent manner (22, 23).

The nucleolus nevertheless remains dynamic in its tripartite organization and is connected to the nuclear matrix by intermediate filament (IF) proteins (24, 25). Lamins are type V intermediate filament proteins at the inner nuclear membrane that maintain nuclear architecture (26). B-type lamins (B1 and B2) are expressed in most vertebrate cells, while lamin A/C is expressed predominantly in differentiated cells (27, 28). Although lamins localize primarily to the nuclear periphery, intranuclear pools of lamins modulate chromatin dynamics, splicing, and DNA damage repair (29–32). Lamin A/C and lamin B1 are implicated in maintaining nucleolar structure and function (33–36). However, the role of lamin B2 in nucleolar structure-function modulation remains unclear.

Here we have uncovered a novel role for lamin B2 in modulating nucleolar morphology and function. We show that lamin B2 localizes at the granular component and associates with nucleolar factors such as nucleolin and nucleophosmin (NPM1) in diploid DLD-1 cells. Furthermore, lamin B2 depletion strikingly disrupts nucleolar morphology and upregulates levels of nucleolar transcripts such as the 45S rRNA and intergenic sequence (IGS) transcripts. The upregulated IGS transcripts show an increased colocalization with nucleolin aggregates in the nucleoplasm. Taken together, these studies unravel a novel role for lamin B2 in modulating nucleolar morphology, dynamics, and function.

## RESULTS

### Lamin B2 depletion disrupts nucleolar morphology.

Lamins and SUN1, which are proteins of the nuclear envelope, regulate nuclear structure and function across cell types (37, 38). Interestingly, lamin A/C, lamin B1, and SUN1 also regulate nucleolar structure and function (33, 35). Here we sought to examine the relatively unappreciated role of lamin B2 in the modulation of nucleolar structure and function in diploid colorectal cancer (DLD-1) cells. We selected DLD-1 cells for our experiments owing to their stable and near diploid karyotype of 44 to 46 chromosomes across passages (data not shown). Furthermore, the levels of all three nuclear lamins, i.e., lamins A/C, B1, and B2, are comparable in DLD-1 cells but vary considerably in most other cell types (39–41).

**FIG 1:**
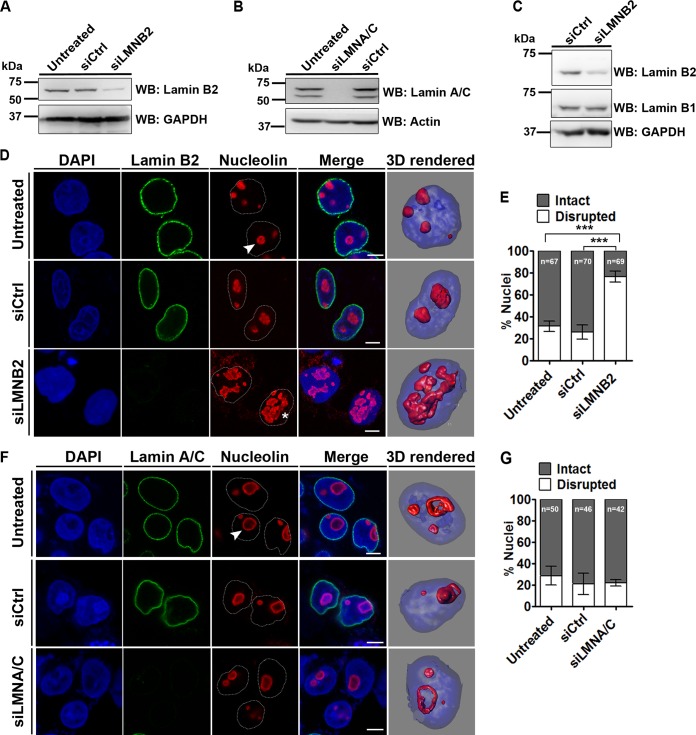
Lamin B2 depletion disrupts nucleolar morphology. (A and B) Western blots (WB) showing siRNA-mediated depletion of lamin B2 (A) and lamin A/C (B). (C) Lamin B1 levels are unaltered upon lamin B2 knockdown. Loading controls were GAPDH (glyceraldehyde-3-phosphate dehydrogenase) and actin. (D) Coimmunostaining of lamin B2 and nucleolin in DLD-1 cells. DAPI counterstains the nucleus. Untreated cells, lamin B2 knockdown cells (siLMNB2), or cells treated with nontargeting siRNA (siCtrl) were used. Representative confocal images of control cells show discrete and intact nucleoli (arrowhead), and siLMNB2 shows disrupted nucleolar morphology (asterisk) (also shown in 3D reconstructions). Scale bars, ∼5 μm. (E) Quantification of nucleolar morphologies shows a significant increase in disrupted nucleoli upon lamin B2 knockdown (***, *P* < 0.001 by Fisher's exact test of proportions) (number of independent biological replicates [*N*] = 3; *n*, number of nuclei). Error bars indicate standard errors of means (SEM). (F) Coimmunostaining of lamin A/C and nucleolin. No change in nucleolar morphology was observed upon siLMNA/C (arrowhead) or siCtrl treatment (also shown in 3D reconstructions). Scale bars, ∼5 μm. (G) Quantification of nucleolar morphologies upon lamin A/C knockdown (*P* > 0.05 by Fisher's exact test of proportions) (*N* = 2; *n*, number of nuclei). Error bars indicate standard deviations (SD).

We performed small interfering RNA (siRNA)-mediated gene silencing of lamins B2 and A/C, followed by immunoblotting assays, which showed ∼70% knockdown of lamin B2 and lamin A/C, independently, in DLD-1 cells (Fig. 1A and B). Since the depletion of the closely related lamin B1 disperses nucleoli in HeLa cells, we ascertained that lamin B2 depletion does not alter the levels of lamin B1 in DLD-1 cells (Fig. 1C) (35). We next examined nucleolar morphologies upon lamin depletion by immunostaining cells with nucleolin, a bona fide nucleolar protein (Fig. 1D and F). Nucleolin localizes at the granular component (GC) of each nucleolus and serves as a marker of nucleolar morphology and numbers across cell types (19, 42). Immunostaining of nucleolin in DLD-1 cells showed two contrasting nucleolar morphologies: (i) intact, i.e., discrete, spatially separate, and spherical nucleoli (Fig. 1D, arrowhead), and (ii) disrupted, i.e., aggregated and irregular nucleoli (Fig. 1D, asterisk). We typically detected 2 or 3 discrete, spherical nucleoli in each nucleus in ∼69% of the cells, while ∼31% of the cells showed disrupted nucleoli (Fig. 1E). Interestingly, lamin B2 depletion revealed a significant increase in strikingly aberrant and disrupted nucleoli in ∼76% of the cells (Fig. 1D [asterisk] and E). In marked contrast, lamin A/C knockdown did not affect nucleolar morphologies (intact, ∼80% of the cells; disrupted, ∼20% of the cells) (Fig. 1F and G). We also employed two independent siRNA oligonucleotides to knock down lamin B2, which showed comparable extents of disrupted nucleoli in DLD-1 cells (siLMNB2.2, ∼59%; siLMNB2.3, ∼62%) (data not shown). Furthermore, nucleolar volumes showed a significant increase (∼1.5-fold) upon lamin B2 but not lamin A/C depletion, consistent with the large-scale nucleolar disruptions that we detected in lamin B2-depleted cells (data not shown). Lamin B2 knockdown, however, did not alter nucleolin levels (data not shown). Taken together, these results underscore a novel role for lamin B2 in the maintenance of discrete and intact nucleolar morphologies.

### Distinct domains of lamin B2 modulate nucleolar and nuclear morphologies.

**FIG 2:**
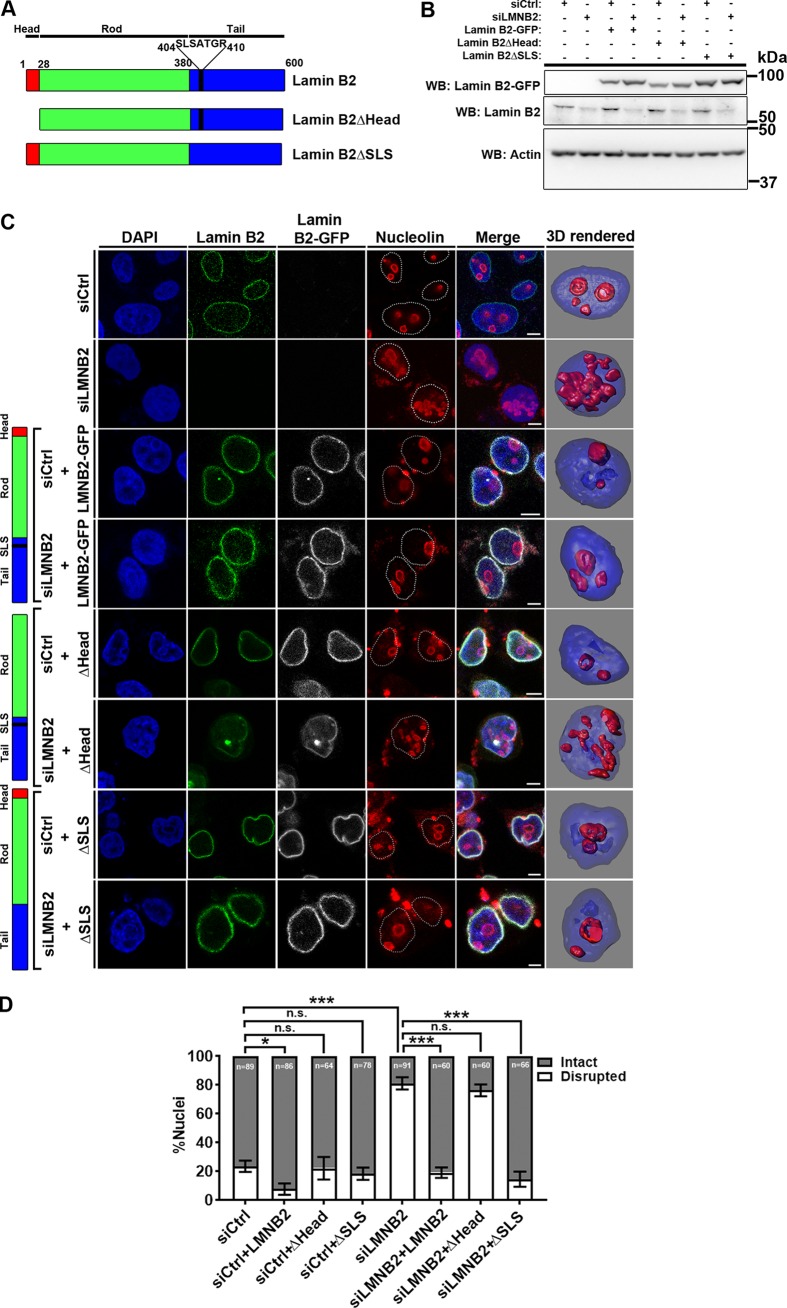
The lamin B2 head domain maintains nucleolar morphology. (A) Schematic representation of full-length lamin B2 with the N-terminal head domain, central rod domain, and C-terminal tail domain. The lamin B2ΔHead mutant lacks the head domain (aa 1 to 28) from the N terminus. The lamin B2ΔSLS mutant lacks the sequence SLSATGR (aa 404 to 410) from the tail domain of lamin B2. (B) Western blot showing overexpression of siRNA-resistant full-length lamin B2-GFP, lamin B2ΔHead-GFP, and lamin B2ΔSLS-GFP mutants in control and lamin B2-depleted cells. The loading control was actin. (C) Immunofluorescence staining of nucleolin, showing the nucleolar morphology in control and lamin B2-depleted cells overexpressing (i) full-length lamin B2-GFP (+LMNB2), (ii) lamin B2ΔHead (+ΔHead), and (iii) lamin B2ΔSLS (+ΔSLS) constructs. Scale bars, ∼5 μm. (D) Intact nucleolar morphology was restored upon overexpression of full-length lamin B2 (siLMNB2+LMNB2) and lamin B2ΔSLS (siLMNB2+ΔSLS) but not upon overexpression of lamin B2ΔHead (siLMNB2+ΔHead). (*, *P* < 0.05; ***, *P* < 0.001; n.s., not significant [by Fisher's exact test of proportions]) (number of independent biological replicates [*N*] = 3; *n*, number of nuclei). Error bars indicate SEM.

To dissect potential separation of the function of lamin B2 in modulating nucleolar and nuclear morphologies, we targeted two amino acid sequences in the N- and C-terminal domains of lamin B2. Lamin B2 is organized as (i) an N-terminal globular head domain (amino acids [aa] 1 to 28), (ii) a central rod domain (aa 29 to 380), and (iii) a C-terminal tail domain (aa 381 to 600) (Fig. 2A) (43, 44). We created two independent deletion mutants of lamin B2: (i) lamin B2ΔHead (with the first 28 amino acids of lamin B2 deleted), required for the head-tail organization of lamin B2 dimers (45), and (ii) lamin B2ΔSLS, with a 7-amino-acid deletion of the SLSATGR sequence from the C-terminal tail domain (Fig. 2A). This amino acid stretch is unique to lamin B2 and absent in lamin A/C and lamin B1.

We determined the effects of overexpressing siRNA-resistant (i) full-length lamin B2, (ii) lamin B2ΔHead, and (iii) lamin B2ΔSLS on the nucleolar and nuclear morphologies in lamin B2-depleted cells. Immunoblotting showed that the expression levels of full-length lamin B2 and the deletion mutants were comparable in control and lamin B2-depleted cells (Fig. 2B). We next expressed full-length lamin B2 and its deletion mutants in DLD-1 cells and examined the nucleolar morphology by nucleolin staining (Fig. 2C).

We first examined the effect of overexpressing full-length siRNA-resistant lamin B2 on nucleolar morphology in lamin B2-depleted cells. Overexpression of full-length lamin B2 reduced the number of disrupted nucleoli in control cells. Disrupted nucleoli induced upon lamin B2 knockdown were also rescued to intact nucleoli upon overexpression of lamin B2 in ∼81% of the cells (Fig. 2C and D). Taken together, these data suggest that an intact nucleolar morphology was rescued upon the restoration of lamin B2 levels.

Transfection of the deletion constructs of lamin B2 did not alter the nucleolar morphology in control cells (Fig. 2C and D). Notably, the disrupted nucleolar morphology in lamin B2-depleted cells was not restored to intact nucleoli upon overexpressing the lamin B2 mutant lacking the head domain (Fig. 2C and D), while overexpression of the lamin B2 mutant lacking the SLSATGR amino acid sequence at the tail domain restored intact nucleolar morphology in ∼85% of the cells (comparable to the case for full-length lamin B2) (Fig. 2C and D), suggesting that the SLSATGR sequence is dispensable for the maintenance of intact nucleoli. Taken together, the results show that the head domain of lamin B2 is required for maintaining intact nucleolar morphologies.

**FIG 3:**
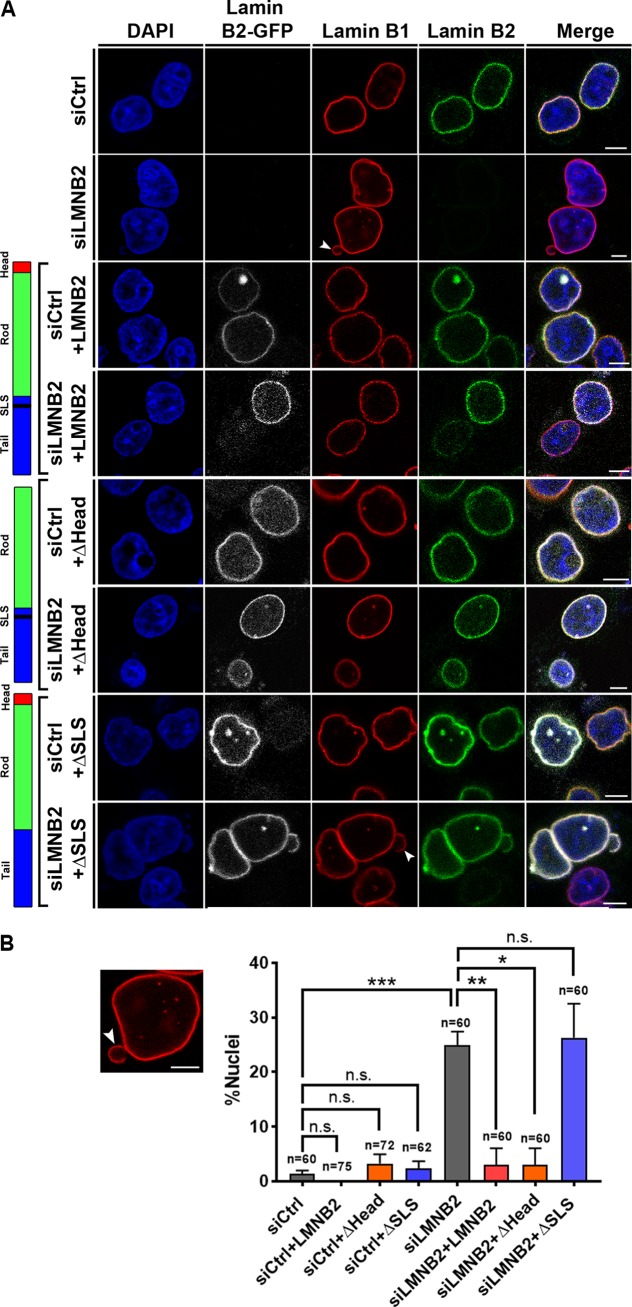
The SLSATGR sequence in the lamin B2 tail domain maintains nuclear morphology. (A) Immunofluorescence staining of lamin B1, showing the nuclear morphology in control and lamin B2-depleted cells overexpressing full-length lamin B2-GFP (+LMNB2), (ii) lamin B2ΔHead (+ΔHead), and (iii) lamin B2ΔSLS (+ΔSLS) constructs. Control cells show ellipsoidal nuclei with uniform lamin B1 staining at the nuclear periphery. Lamin B2-depleted cells show nuclear blebs that partially stain for lamin B1 (arrowhead). Scale bars, ∼5 μm. (B) The incidence of nuclear blebs in lamin B2 depleted cells was reduced upon overexpression of full-length lamin B2 (siLMNB2+LMNB2) and lamin B2ΔHead (siLMNB2+ΔHead) but not upon overexpression of lamin B2ΔSLS (siLMNB2+ΔSLS). (*, *P* < 0.05; **, *P* < 0.01; ***, *P* < 0.001 [by Student's *t* test]) (number of independent biological replicates [*N*] = 3; *n*, number of nuclei). Error bars indicate SEM.

Lamin B2 knockout induces severe defects in the nuclear envelope (37). We were curious to determine if lamin B2 mutants exert mutually exclusive effects on the nucleolar and nuclear morphologies. We examined the effect of lamin B2 depletion on nuclear morphology. Confocal imaging of DLD-1 cells immunostained for lamin B1 consistently revealed a homogenous population of ellipsoidal nuclei, with a regular and uniform nuclear envelope (Fig. 3A, siCtrl). A small subpopulation (∼1%) of control cells, however, showed nuclear blebs (Fig. 3B). Remarkably, lamin B2 knockdown induced the formation of nuclear blebs in ∼25% of DLD-1 cells (Fig. 3A [siLMNB2, arrowhead] and B). Overexpression of full-length lamin B2 decreased nuclear blebs from ∼25% to ∼3% in lamin B2-depleted cells (Fig. 3B, siLMNB2+LMNB2), while overexpression of lamin B2ΔHead also reduced nuclear blebs to ∼3% (Fig. 3B, siLMNB2+ΔHead). Overexpression of the lamin B2ΔSLS mutant, in contrast, did not reduce the extent of nuclear bleb formation in lamin B2-depleted cells (Fig. 3B, siLMNB2+ΔSLS), suggesting that the SLSATGR sequence is indeed required for maintaining the normal and bleb-free nuclear morphology, while the head domain of lamin B2 is dispensable for the same. We also found that overexpression of full-length lamin B2, lamin B2ΔHead, or lamin B2ΔSLS did not induce nuclear blebs in control DLD-1 cells (Fig. 3B). In summary, the analyses of lamin B2 mutants was revealing at the mechanistic level, as this unraveled a distinct separation of function of lamin B2 in independently modulating nucleolar and nuclear morphologies.

### Lamin B2 localizes at the nucleolar border and associates with nucleolin and nucleophosmin.

It is well established that lamin B2 localizes primarily at the inner nuclear membrane across cell types (46, 47). Lamin B2 staining in intact nuclei shows hardly any intranuclear localization (Fig. 1D). We therefore permeabilized the nucleus by salt extractions and DNase I treatment in order to facilitate antibody accessibility into the nuclear matrix (48, 49). This approach revealed a distinct intranuclear localization of lamin B2 and lamin A/C inside the nucleus. Notably, lamin B2 but not lamin A/C localized proximal to the nucleolar border (data not shown).

**FIG 4:**
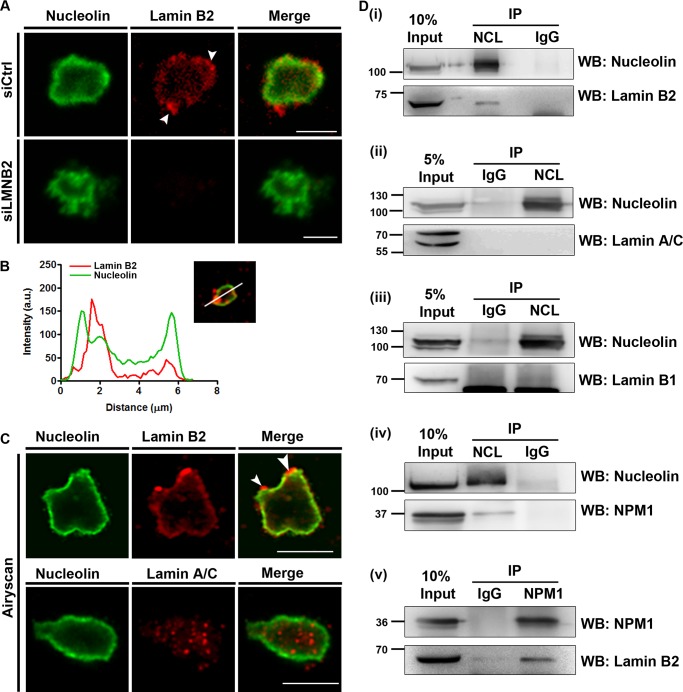
Lamin B2 associates with the nucleolus. (A) Isolated nucleoli were immunostained for lamin B2 and nucleolin. Nucleolin localizes largely at the nucleolar edge. Lamin B2 foci were enriched at the nucleolar periphery (siCtrl panel, arrowheads). The absence of lamin B2 staining in nucleoli isolated from lamin B2-depleted cells (siLMNB2 panel) shows the specificity of lamin B2 staining at the nucleolus. Scale bars, ∼5 μm. (B) A representative line scan across an isolated nucleolus shows lamin B2 (red line) enrichment near the edge of the nucleolus marked by nucleolin (green line). (C) Superresolution Airyscan images of isolated nucleoli immunostained for nucleolin, lamin B2, and lamin A/C. Lamin B2 is enriched at the nucleolar border (arrowheads), while lamin A/C foci localize in the nucleolar interior. Scale bars, ∼5 μm. (D) (i) Coimmunoprecipitation of lamin B2 with nucleolin (NCL). Negative control, IgG. (ii and iii) Lamin A/C (ii) and lamin B1 (iii) do not coimmunoprecipitate with nucleolin. (iv) Nucleolin pulls down NPM1, which serves as a positive control. (v) Lamin B2 coimmunoprecipitates with NPM1. Co-IP experiments were performed in three independent biological replicates for lamin B2 and two independent biological replicates for lamin A/C and lamin B1.

To determine if lamin B2 indeed associates with the nucleolus, we performed immunofluorescence staining of lamin B2 on isolated nucleoli from a semiconfluent culture of DLD-1 cells (Fig. 4A). Remarkably, isolated nucleoli showed a distinct localization and enrichment of lamin B2 at the nucleolar border as foci in close proximity to nucleolin (Fig. 4A [siCtrl, arrowheads] and B [line scan]). Nucleoli isolated from lamin B2-depleted cells did not show lamin B2 staining, while nucleolin staining was maintained at the nucleolar border (Fig. 4A, siLMNB2). This further validated the specificity of lamin B2 localization at the nucleolar border in isolated nucleoli.

We also performed superresolution microscopy using Airyscan imaging to further resolve lamin B2 and lamin A/C localization within isolated nucleoli (Fig. 4C). This high-resolution imaging approach recapitulated lamin B2 localization at the nucleolar border (Fig. 4C). In contrast, lamin A/C showed a punctate distribution in the nucleolar interior, while nucleolin localization was confined to the nucleolar border (Fig. 4C). The nucleolar localization of lamin A/C is consistent with its detection in nucleolar extracts of HeLa cells by mass spectrometry (35). Although confocal and superresolution Airyscan imaging showed a close proximity of lamin B2 with nucleolin (Fig. 4B, line scans), we did not detect a colocalization between lamin B2 and nucleolin at all regions of isolated nucleoli.

Considering the proximity of lamin B2 to nucleolin, we asked if lamin B2 associates with bona fide nucleolar proteins. We performed coimmunoprecipitation (co-IP) assays with nucleolin and nucleophosmin (NPM1) on whole-cell extracts of DLD-1 cells (Fig. 4D). Coimmunoprecipitation of nucleolin specifically immunoprecipitated lamin B2 but not lamin A/C or lamin B1 (Fig. 4D, panels i to iii). Under similar conditions, we recapitulated the well-established interaction between nucleolin and NPM1 (50) (Fig. 4D, panel iv). Lamin B2 also coimmunoprecipitated with NPM1, underscoring the association of lamin B2 with nucleolar proteins at the granular component (Fig. 4D, panel v). In summary, these results strongly implicate lamin B2 in the structural and potentially functional organization of the nucleolus.

### Lamin B2 depletion enhances nucleolin aggregation in the nucleoplasm.

**FIG 5:**
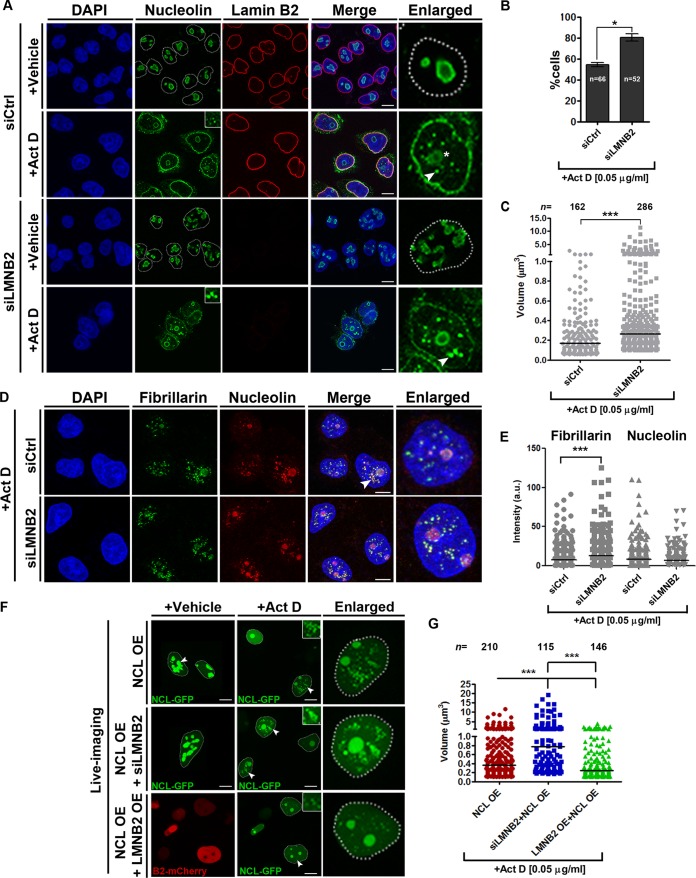
Lamin B2 depletion increases the volume of nucleolin aggregates. (A) Control or lamin B2-depleted cells were treated with DMSO (vehicle control) or actinomycin D (+Act D) and immunostained for nucleolin. Vehicle-treated control (siCtrl) and lamin B2-depleted (siLMNB2) cells show nucleolin restricted to the nucleolus. Vehicle-treated lamin B2-depleted cells show an irregular nucleolar morphology (siLMNB2, +Vehicle panel). Act D-treated control (siCtrl+Act D) or lamin B2-depleted (siLMNB2+Act D) cells show nucleolin aggregates in the nucleoplasm (insets, enlarged panels [arrowhead]). Act D treatment shows spherical nucleoli (asterisk, enlarged panel). Scale bars, ∼5 μm. (B) Lamin B2 depletion shows a significant increase in cells with nucleolin aggregates upon Act D treatment (*, *P* < 0.05 by Student's *t* test) (number of independent biological replicates [*N*] = 3; *n*, number of nuclei). Error bars indicate SEM. (C) Scatter plots showing an increase in the volumes of nucleolin aggregates upon lamin B2 depletion followed by Act D treatment (***, *P* < 0.001 by Mann-Whitney test). Bar, median (*N* = 3; *n*, number of aggregates; siCtrl, 32 nuclei; siLMNB2, 35 nuclei). (D) Fibrillarin colocalizes with nucleolin aggregates upon Act D treatment (arrowhead). Scale bars, ∼5 μm. (E) Lamin B2-depleted cells show a significant increase in fibrillarin intensity within nucleolin aggregates (***, *P* < 0.001 by Mann-Whitney test) (*N* = 2; siCtrl, 28 nuclei; siLMNB2, 30 nuclei). (F) Live imaging of DLD-1 cells overexpressing nucleolin (NCL-GFP OE) following Act D or vehicle treatment. NCL-GFP-transfected cells phenocopy disrupted nucleolar morphology comparably to lamin B2 depletion (NCL-GFP OE, arrowhead). Act D-treated cells show aggregates of nucleolin in the nucleoplasm (arrowhead). Lamin B2-depleted cells overexpressing nucleolin show relatively larger aggregates in the nucleoplasm (NCL OE+ siLMNB2, inset). Cells coexpressing lamin B2-mCherry and NCL-GFP show smaller aggregates, suggesting a rescue of the phenotype of lamin B2 depletion (NCL OE+ LMNB2 OE, inset). Insets, nucleolin aggregates. Scale bars, ∼5 μm. (G) Scatter plots showing volumes of nucleolin aggregates. Lamin B2 depletion significantly increases the volumes of nucleolin aggregates (siLMNB2+NCL OE), while coexpression of lamin B2 and nucleolin (LMNB2 OE + NCL OE) rescues the volume of nucleolin aggregates to basal levels. Bars, median (***, *P* < 0.001 by Mann-Whitney test) (*N* = 3, *n*, number of aggregates; NCL OE, 30 nuclei; siLMNB2+NCL OE, 30 nuclei; LMNB2 OE+NCL OE, 25 nuclei).

Active transcription by RNA polymerase I (Pol I) is essential for the maintenance of nucleolar structure and function. This was corroborated by actinomycin D (Act D)-mediated inhibition of RNA Pol I, which induces reorganization of fibrillarin and UBF into nucleolar caps and dispersal of nucleolin and NPM1 into the nucleoplasm (7). Act D treatment is a useful experimental paradigm to address the effects of potential regulators on the morphology and function of the nucleolus. We determined the effect of lamin B2 depletion on the subnuclear localization of nucleolin and nucleolar morphology upon Act D treatment. Immunostaining of nucleolin in Act D-treated cells consistently showed cells with (i) smaller and hollow nucleoli (Fig. 5A, asterisk), (ii) nucleolar cap formation, and (iii) nucleolin dispersion into the nucleoplasm (Fig. 5A, siCtrl+Act D, arrowhead). Furthermore, nucleolin was enriched at the nuclear periphery, consistent with nucleolin shuttling out of the nucleolus in Act D-treated cells (51). Act D treatment induced nucleolin aggregates in the nucleoplasm (Fig. 5A, siCtrl+Act D, arrowhead). Of note, lamin B2-depleted cells treated with Act D showed a significant increase in cells with nucleolin aggregates (siCtrl, ∼54%; siLMNB2, ∼80%) (Fig. 5B) and also an increase in their volume (∼1.3-fold) (Fig. 5C). Notably, fibrillarin costained with nucleolin aggregates, suggesting the association of nucleolar RNA binding proteins within these aggregates and destabilization of the nucleolus (Fig. 5D). Fibrillarin also showed a relatively higher intensity in these aggregates upon lamin B2 depletion (Fig. 5E).

We determined whether lamin B2 modulates the status of nucleolin aggregates in cells overexpressing nucleolin. Interestingly, nucleolin overexpression phenocopied the disrupted nucleolar morphologies that we consistently detect upon lamin B2 depletion (Fig. 5F, NCL OE + vehicle, arrowhead). Additionally, nucleolin overexpression showed nucleolin aggregates in the nucleoplasm upon Act D treatment (Fig. 5F and G, NCL OE + Act D) (volume [*M*] = 0.36 μm^3^). Lamin B2 knockdown showed a significant increase in the volume of nucleolin aggregates (*M* = 0.77 μm^3^) of ∼2.13-fold, while lamin B2 overexpression rescued the volume of nucleolin aggregates to near-basal levels (*M* = 0.25 μm^3^) (Fig. 5F and G). Taking the data together, we conclude that lamin B2 modulates nucleolin aggregation in the nucleoplasm.

### Nucleolin aggregates persist in the nucleoplasm upon lamin B2 depletion.

**FIG 6:**
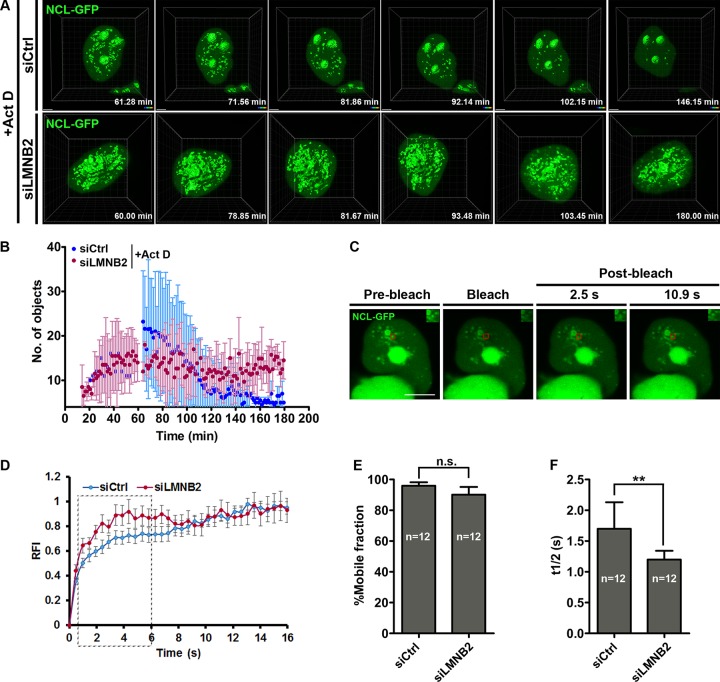
Persistence of nucleolin aggregates upon lamin B2 depletion. (A) Control and lamin B2-depleted cells were transfected with NCL-GFP and treated with Act D. 4D time-lapse confocal imaging shows nucleolin aggregates that peak at ∼1 h after Act D addition and gradually disperse into the nucleoplasm (siCtrl,). In lamin B2-depleted cells, nucleolin aggregates persist for ∼3 h (siLMNB2 panel). Scale bars, ∼2 μm. (B) Quantification of nucleolin aggregates from reconstructions of 4D time-lapse movies, plotted as a function of time (number of independent biological replicates [*N*] = 3; *n* = 6 nuclei each), shows the persistence of nucleolin aggregates upon lamin B2 depletion. (C) Nucleolin aggregates (NCL-GFP) were photobleached to assess nucleolin dynamics. Representative images of nucleolin speckles from control cells are shown. Red boxes, bleach ROI. Insets, photobleached ROI. Scale bar, ∼5 μm. (D) FRAP curve shows recovery of nucleolin in aggregates from lamin B2-depleted and control cells. Dashed box, initial phase of recovery is faster upon lamin B2 depletion. (E) The mobile fraction of nucleolin calculated from panel C is not altered upon lamin B2 depletion. Error bars, SEM (*P* > 0.05 by Student's *t* test) (*N* = 3; *n*, number of nuclei). (F) Nucleolin recovery is significantly faster upon lamin B2 depletion (**, *P* < 0.01 by Student's *t* test) (*N* = 3; *n*, number of nuclei).

We determined the effect of lamin B2 depletion on the dynamics of nucleolin aggregates by live-cell imaging. This revealed a progressive increase in nucleolin aggregates originating from the nucleolus, from ∼40 min after Act D addition (Fig. 6A [siCtrl] and B; see Movie S1 in the supplemental material). In control cells, nucleoplasmic aggregates of nucleolin showed a steady decline and were hardly detectable after ∼2 h, suggesting a dispersal of nucleolin into the nucleoplasm (Fig. 6A and B [blue circles]). Remarkably, nucleolin aggregates in lamin B2-depleted cells persisted in the nucleoplasm even after ∼3 h of Act D treatment (Fig. 6A and B [red circles]; see Movies S2 and S3 in the supplemental material). This suggests that lamin B2 depletion promotes the long-term retention of nucleolin aggregates in the nucleoplasm. We performed fluorescence recovery after photobleaching (FRAP) to examine whether lamin B2 regulates nucleolin dynamics within the aggregates (Fig. 6C and D). Nucleolin showed ∼95% recovery, suggesting a free exchange of nucleolin into the aggregates (Fig. 6D and E). This is consistent with the free diffusion of nucleolin in the nucleoplasm of HeLa cells treated with Act D (52). Although the relative mobile fractions of nucleolin within the aggregates were comparable (Fig. 6E), nucleolin recovery was significantly faster upon lamin B2 depletion (half-life [*t*_1/2_], ∼1.2 s) than that for control cells (*t*_1/2_, ∼1.7 s) (Fig. 6F). Taken together, these results suggest an increased recruitment of nucleolin into the aggregates upon lamin B2 depletion.

### Lamin B2 depletion increases rRNA expression.

**FIG 7:**
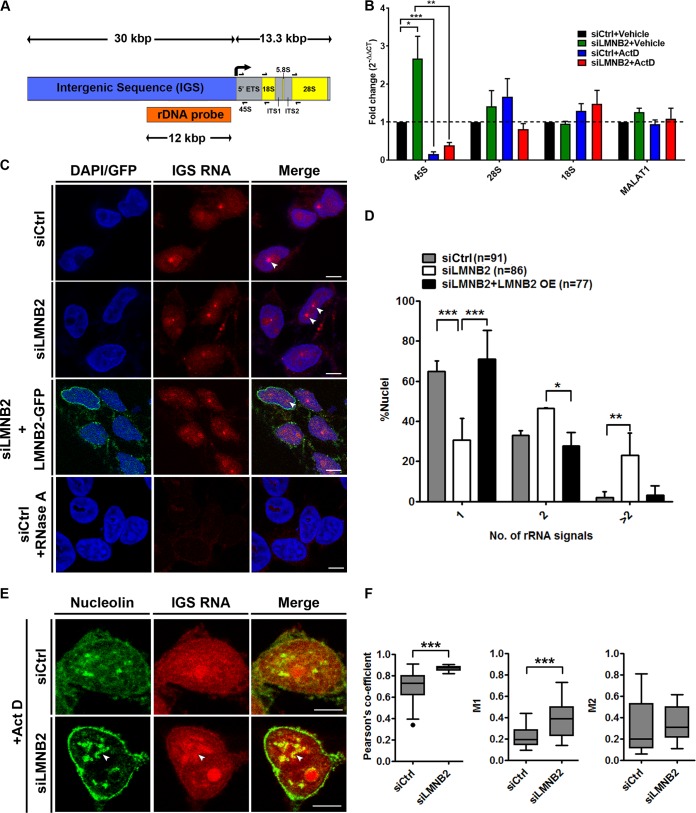
Lamin B2 depletion upregulates expression levels of nucleolar transcripts. (A) Schematic representation of the rDNA encoding the ∼13.3-kbp 45S rRNA and ∼30-kbp intergenic sequence. The primer pairs used for qRT-PCR (half arrows) and the ∼12-kbp probe for RNA-FISH are indicated. (B) qRT-PCR shows a significant increase in 45S transcript levels upon lamin B2 depletion. Act D treatment significantly reduces expression levels of the 45S rRNA transcript in both control and lamin B2-depleted cells. Lamin B2 depletion does not show a significant change in the levels of 28S and 18S transcripts (number of independent biological replicates [*N*] = 3). MALAT1 expression levels are not altered upon lamin B2 depletion and serve as a negative control (*N* = 3; *, *P* < 0.05; **, *P* < 0.01; ***, *P* < 0.001 [by Student's *t* test]). (C) RNA-FISH labels intergenic sequence (IGS) RNA in the nucleolus. Lamin B2-depleted cells show amplification of IGS RNA (siLMNB2 panel, arrowhead). Overexpression of siRNA-resistant lamin B2-GFP in lamin B2-depleted cells (siLMNB2+LMNB2-GFP) restores the number of IGS RNA-FISH signals. The absence of RNA signals in cells upon RNase A treatment shows specificity of IGS RNA-FISH foci. Scale bars, ∼5 μm. (D) Quantification of RNA-FISH foci shows a significant increase (>2 foci) upon lamin B2 depletion, while overexpression of lamin B2 restores IGS transcripts to a single focus. Error bars, SD. (*, *P* < 0.05; **, *P* < 0.01; ***, *P* < 0.001 [by Fisher's exact test of proportions]) (*N* = 3, *n*, number of nuclei). (E) Immuno-RNA-FISH shows colocalization of nucleolin speckles with IGS transcripts in control or lamin B2-depleted cells treated with Act D (arrowhead). (F) Increased colocalization of IGS transcripts with nucleolin in the nucleoplasm upon lamin B2 knockdown (Pearson colocalization index median values: control, 0.7; siLMNB2, 0.88). Manders coefficient (M1), overlap of IGS RNA with nucleolin (median values; control, 0.19; siLMNB2, 0.38). Manders coefficient (M2), overlap of nucleolin with IGS RNA (median values: control, 0.2; siLMNB2, 0.31 [not significant]). Whiskers, Tukey (***, *P* < 0.001 by Mann-Whitney test) (*N* = 3; siCtrl, 28 nuclei; siLMNB2, 32 nuclei).

Lamin B2 depletion significantly disrupts the nucleolar morphology and affects the dynamics of nucleolin aggregates (Fig. 1D and E and 6D to F). We determined whether lamin B2 also modulates nucleolar function by monitoring the levels of nucleolus-specific transcripts. We examined the expression levels of key nucleolar transcripts (by quantitative real-time PCR [qRT-PCR]) from two independent regions of the rDNA repeat cluster, i.e., (i) 45S rRNA, an ∼13-kbp rRNA coding region which is further processed into 28S, 5.8S, and 18S rRNAs, and (ii) the intergenic sequence (IGS), an ∼12-kbp region upstream of the 45S rRNA start site, by RNA fluorescent *in situ* hybridization (RNA-FISH) (Fig. 7A). The rDNA IGS encodes noncoding RNAs (ncRNAs) such as promoter-associated RNAs (pRNAs) and promoter and pre-rRNA antisense (PAPAS) in response to serum starvation, heat shock, or acidosis (53–55). Interestingly, these ncRNAs regulate 45S rRNA expression, or sequester chaperones (heat shock proteins [HSPs] and Von Hippel-Lindau tumor suppressor protein [VHL]) into the nucleolus (54, 55).

Remarkably, lamin B2 depletion showed a significant increase in the levels of the RNA Pol I-transcribed 45S rRNA (fold change, ∼2.7), while the long noncoding MALAT1 RNA (transcribed by RNA Pol II) was unaffected (Fig. 7B). Taken together, these findings reveal a unique role for lamin B2 and underscores its specificity in upregulating 45S rRNA levels. The expression levels of 45S rRNA showed a significant decline (∼84% decrease) upon Act D treatment (Fig. 7B, siCtrl+Act D). Although Act D treatment downregulated 45S rRNA levels in control cells by ∼84%, the decrease in 45S rRNA levels was only ∼61% in lamin B2-depleted cells. This reiterates that the reduction in lamin B2 levels counters the effect of Act D in downmodulating the levels of 45S rRNA (Fig. 7B). Levels of 28S and 18S processed rRNA transcripts were not significantly altered upon lamin B2 depletion or Act D treatment, suggesting that lamin B2 depletion may not affect processing of 45S pre-rRNA (Fig. 7B).

Having detected a specific increase in 45S rRNA levels, we examined the expression levels and nuclear localization of IGS RNA by RNA-FISH upon lamin B2 depletion (Fig. 7C). RNA-FISH revealed three subpopulations of cells showing IGS foci: (i) a single focus (∼65% of the cells), (ii) 2 foci (∼33% of the cells), and (iii) >2 foci (∼2% of the cells) (Fig. 7D, gray bars). Lamin B2 depletion showed a marked decrease in cells with a single focus (∼31% of the cells) and a concomitant increase in cells with 2 foci (∼46% of the cells) and >2 foci (23% of the cells) (Fig. 7D, white bars). Taking the results together, in addition to the 45S rRNA, lamin B2 also upregulates expression levels of nucleolus-specific intergenic transcripts. Remarkably, lamin B2 overexpression rescued levels of IGS RNA signals to a single focus (∼71%), comparable to the case for control cells (Fig. 7D, black bars).

We next examined the nuclear localization of IGS RNA upon Pol I inhibition (Fig. 7E). In contrast to the distinct foci of IGS RNA signals in untreated cells (Fig. 7C), IGS RNA localization was relatively diffuse upon Act D treatment. Furthermore, IGS RNA colocalized with nucleolin aggregates upon Act D treatment (Fig. 7E). IGS RNA and nucleolin colocalization was enhanced in lamin B2-depleted cells (Fig. 7E and F). In summary, lamin B2 depletion promotes the stability of nucleolin-IGS RNA aggregates in the nucleoplasm. We conclude that lamin B2 functions as a key modulator of nucleolar morphology, dynamics, and function.

## DISCUSSION

**FIG 8:**
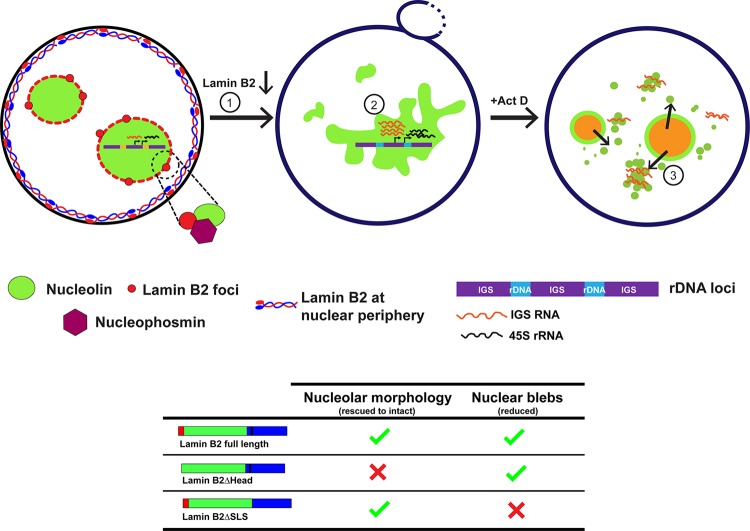
Model depicting a role for lamin B2 in modulating nucleolar structure and function. Lamin B2 is localized predominantly at the nuclear periphery. However, lamin B2 also localizes at the nucleolar border and potentially interacts with nucleolin and NPM1. Lamin B2 depletion shows disrupted nucleolar morphology (1) and increased expression of 45S rRNA and intergenic sequence RNA (IGS RNA) (2). Lamin B2-depleted cells treated with actinomycin D (Act D) have increased nucleolin-IGS RNA aggregates that persist in the nucleoplasm (3). The lamin B2 head domain is required for the maintenance of intact nucleolar morphology, while the lamin B2 SLSATGR amino acid sequence is required for maintaining bleb-free nuclei.

It is well established that lamins are required for the maintenance of nuclear architecture and function (56). Here we show that in addition to providing mechanical and structural integrity to the nucleus (37), lamin B2, and its head domain in particular, is required for maintaining the intact and discrete morphology of the nucleolus. Lamin B2 potentially exerts its nucleolar function by localizing at the nucleolar border and by associating with nucleolar factors such as nucleolin and nucleophosmin. Furthermore, lamin B2 modulates the extent of nucleolin aggregation and dynamics in the nucleus. Lamin B2 depletion strikingly increases the expression levels of key nucleolus-specific transcripts such as the 45S rRNA and the upstream IGS RNA (Fig. 8). Taken together, the results of this study unravel a novel role for nuclear lamin B2 in the maintenance of nucleolar structure and function.

Although the nucleus does not have membrane-bound subcompartments, the nucleolus achieves a remarkable level of compartmentalization by phase separation through nucleolar proteins such as nucleophosmin and fibrillarin, which also maintain the relatively spherical morphology of the nucleolus (22, 23, 57). Nucleolin and NPM1 have alternate stretches of basic and acidic amino acid residues at the N terminus, which are likely to function in the phase separation of the nucleolus. We surmise that given the uniquely peripheral localization of lamin B2 at the nucleolar border (Fig. 4), lamin B2 may associate with the negatively charged regions of nucleolin and NPM1 and further assist them in phase separating the nucleolus. It is evident that the loss of lamin B2 disorganizes the nucleolus and enhances the formation of nucleolin aggregates in the nucleoplasm, further implicating lamin B2 in modulating nucleolar phase separation (Fig. 1D and 5A). The contribution of the cytoskeleton in conjunction with nuclear lamins and LINC complex proteins in phase separating nuclear suborganelles is an active area of investigation. The nuclear membrane proteins SUN1, lamin A/C, and lamin B1 regulate nucleolar structure and function in human mammary epithelial MCF10A cells and in HeLa cells (33, 35). Further, the disruption of F-actin filaments by latrunculin treatment results in nucleolar aggregates in Xenopus germinal vesicle nuclei. This suggests that nucleoskeletal tension between the nucleolus and nuclear matrix potentially contributes to the discreteness of individual nucleoli (58). Of note, lamin A/C knockdown led to nucleolar expansion in MCF10A cells and fibroblasts (33, 34), while lamin B1 depletion in HeLa cells resulted in nucleolar dispersal (35). This suggests that lamin function in the regulation of nucleolar structure and function is cell type specific.

### Lamin B2 mutants exert mutually exclusive effects on nucleolar and nuclear morphologies.

Lamins form structured polymers at the nuclear membrane as revealed by cryo-electron tomography and three-dimensional structured illumination microscopy (3D-SIM) (46, 59). Lamins homo- or heterodimerize *in vitro* via their rod domains (45). Lamin dimers form head-to-tail parallel polymers, which laterally associate to form stacks of lamin filaments (60). We surmise that the lamin B2 organization at the nuclear periphery is strikingly different from that at the nucleolar border, owing to its stable association with lamin A/C and B1 at the nuclear periphery (Fig. 4). The head domain of lamin B2 is required for the head-to-tail polymerization of lamin B2 dimers. Head domain deletion mutants of chicken lamin B2 and mouse lamin A do not form head-to-tail polymers *in vitro* (60, 61). However, in mammalian cells, lamin AΔHead did not localize at the nuclear periphery and showed nuclear aggregates, while lamin B1ΔHead correctly localized to the nuclear periphery, with no effect on endogenous lamins (62). This suggests the differential localization and function of the head domains of A-type and B-type lamins at the nuclear envelope. Interestingly, our studies suggest that the head domain of lamin B2 is required for maintaining nucleolar morphology but not nuclear morphology (Fig. 2 and 3).

It is possible that lamin B2 forms head-to-tail polymers at the nucleolar periphery in order to maintain the spherical morphology and discrete organization of the nucleolus. A possibility remains that the lamin B2ΔHead mutant heterodimerizes with endogenous lamin A/C or lamin B1 via its rod domain at the nuclear envelope, thereby rescuing the extent of nuclear blebs (63).

Nuclear rupture and nuclear blebs during cancer cell migration are enhanced upon lamin B2 depletion (64). Consistent with previous reports, nuclear blebs were enriched in regions of the nuclear membrane with reduced lamin B1 levels (Fig. 3B, arrowhead) (64). The tail domains of lamins with the CAAX motif and IgG fold are required for their integration into the nuclear membrane and interaction with emerin and histones (65–67). It is interesting that the relatively uncharacterized and unique amino acid stretch SLSATGR in the C-terminal region of lamin B2 modulates the formation of nuclear blebs (Fig. 3). This suggests that the SLSATGR sequence is required for the proper organization of the nuclear lamina, as its absence increases the propensity for nuclear blebs.

### Lamin B2 associates with the nucleolus and interacts with nucleolar factors.

The nucleolar localization sequence (NoLS) guides the localization of several ribosomal and nonribosomal proteins, such as human telomerase reverse transcriptase (hTERT) and fibroblast growth factor 3 (FGF-3), in the nucleolus (68–70). In addition to the NoLS, c-Myc protein for instance is recruited to the nucleolus via its interaction with NPM1 (71). Furthermore, NPM1 phase-separates and integrates into the nucleolus by interacting with RXn1R motif-containing proteins (22). Interestingly, we found several RXn1R motifs in lamin B2 that may facilitate its nucleolar localization. We propose that lamin B2 localized at the GC compartment functions as a barrier (Fig. 4A) and limits nucleolin mobility in and out of the nucleolus, since lamin B2 depletion was associated with increased rates of nucleolin recovery into nucleolin aggregates in the nucleoplasm (Fig. 6F). Nucleolin stability within the nucleolus is important, as its nucleoplasmic localization is associated with the degradation of p53 mRNA (72, 73).

### Lamin B2 modulates nucleolin aggregation.

The formation of interphase prenucleolar bodies (iPNBs) associated with unprocessed rRNA is induced in cells under hypotonic stress (74). Here, we show that the inhibition of RNA Pol I by Act D treatment induces the aggregation of nucleolin speckles in the nucleoplasm, which is further enhanced upon lamin B2 depletion (Fig. 5A and F). We envisage three potential mechanisms by which loss of lamin B2 enhances nucleolin stability, i.e., (i) promoting self-oligomerization of nucleolin, (ii) stable association of nucleolin with other nucleolar proteins such as fibrillarin (Fig. 5D), or (iii) nucleolin-RNA complex formation (Fig. 7E), collectively resulting in the prolonged stability of nucleolin aggregates (72, 75).

Cellular stresses such as acidosis are associated with the expression of noncoding RNAs from intergenic rDNA sites that sequester stress-responsive proteins such as HSP70 and VHL in the nucleolus (55). An increase in the expression levels of IGS RNA in lamin B2-depleted cells is suggestive of cellular stress (Fig. 7C and D). The enhanced colocalization of nucleolin with IGS RNA upon Act D treatment in lamin B2-depleted cells suggests the stabilization of the nucleolin-IGS RNA complex (Fig. 7E and F). This is consistent with the faster recovery of nucleolin into the aggregates (Fig. 6F), potentially due to its affinity to IGS RNA. Nucleolin mobility and sequestration into the nucleolus are also modulated by ncRNAs such as Alu RNA and intergenic RNAs expressed upon acidosis (75). We surmise that the RNA binding domains of nucleolin regulate nucleolin-RNA dynamics in a lamin B2-dependent manner (76).

### Lamin B2 regulates rRNA expression.

rRNA expression is stringently regulated in cells (77, 78). Our assays reveal that 45S rRNA expression levels are elevated upon lamin B2 depletion (Fig. 7B). However, lamin B2 depletion also enhances expression levels of IGS RNA (Fig. 7C and D). Therefore, the regulatory role of lamin B2 also extends to the upstream regions of pre-rRNA and is not limited to the pre-rRNA promoter. A tempting possibility is the decondensation of rDNA clusters in the absence of lamin B2, which otherwise associate with heterochromatin binding factors such as HP1 and MacroH2A (79, 80). Consistent with these findings, the loss of MacroH2A.1 disrupts nucleoli and upregulates 45S rRNA, effects that are similar to those of lamin B2 depletion (80–82). It is conceivable that lamin B2 may modulate the recruitment of nucleolin or MacroH2A.1, which are enriched on the rDNA promoter regions as revealed by chromatin immunoprecipitation (ChIP) (83). Effectively, the concerted association of lamin B2 with activators of rDNA transcription such as nucleolin or repressors such as MacroH2A is likely to regulate rDNA transcription.

Our studies reveal a novel and unique role for lamin B2, and its head domain in particular, in maintaining nucleolar structure. Lamin B2 depletion therefore modulates nucleolar morphology, alters nucleoplasmic dynamics of nucleolin, and elevates nucleolar transcripts (Fig. 8). Taken together, our studies implicate lamin B2 as a unique orchestrator of the structural and functional organization of the nucleolus.

## MATERIALS AND METHODS

### Cell lines, cell culture, and transfections.

DLD-1 colorectal adenocarcinoma cells (a gift from Thomas Ried, NCI/NIH, USA), were grown in RPMI medium (Gibco, 11875) supplemented with penicillin (100 units/ml)-streptomycin (100 μg/ml) (Gibco, 15070-063) and 10% heat-inactivated fetal bovine serum (FBS) (Gibco, 6140). Cells were cultured at 37°C in the presence of 5% CO_2_. We repeatedly validated DLD-1 cells across passages by karyotyping and consistently found near-diploid chromosome numbers (44–46) (data not shown). We ensured that all cultures were free of Mycoplasma.

Transient siRNA transfections were performed using Lipofectamine RNAimax reagent (Invitrogen, 13778) in reduced serum Opti-MEM (Gibco, 31985) for 6 h, after which cells were transferred to complete medium and incubated for 48 h. Cells were transfected with plasmids using Lipofectamine LTX with Plus reagent (Invitrogen, 15338-100) or Trans-IT 2020 (Mirus). Green fluorescent protein (GFP)-nucleolin, lamin B2-mCherry, and lamin B2-GFP constructs were kind gifts from Sui Huang and T. Tomonaga (41, 52). Lamin B2 ΔHead and ΔSLS deletion mutants were generated from the lamin B2-GFP construct by two consecutive PCRs and cloned into the pEGFP-N1 vector. The siRNA-resistant lamin B2-GFP plasmids was generated by site-directed mutagenesis (SDM). For experiments requiring both siRNA and plasmid DNA, transfections were performed sequentially; siRNA transfection was performed as mentioned above, DNA transfections were performed after 24 h, and cells were processed after 48 h. The siRNAs used are listed in Table 1.

**TABLE 1:** siRNAs and primers used in this study

siRNA or primer(s)	Sequence(s)
siRNAs	
siLMNB2	5′-GAGCAGGAGAUGACGGAGA-3′
siLMNA/C	5′-CAGUCUGCUGAGAGGAACA-3′
siCtrl1 (LMNB2 scramble)	5′-GGAAGCGUAGACGGAAGAG-3′
siCtrl2 (LacZ)	5′-CGUACGCGGAAUACUUCGA-3′
siCtrl3 (LMNA/C scramble)	5′-GGAGGUCGAGCCAAUAUCA-3′
siLMNB2.2	5′-CCAAGAAGAGGGAGGGCGA-3′
siLMNB2.3	5′-GGAAGAGUGUGUUCGAGGA-3′
Primers	
SDM primer for generating si*LMNB2-GFP	Sense, 5′-ATGCTGGACGCCAAGGAACAAGAAATGACAGAAATGCGGGACGTGATGCA-3′; antisense, 5′-TGCATCACGTCCCGCATTTCTGTCATTTCTTGTTCCTTGGCGTCCAGCAT-3′
Primers for generating lamin B2ΔHead mutant	Sense, 5′-CGAGCTCAAGCTTATATGGAGCTGCGCGAGC-3′; antisense, 5′-GCTCGCGCAGCTCCATATAAGCTTGAGCTCG-3′
Primers for generating lamin B2ΔSLS mutant	Sense, 5′-GCAGCAGCGGCCTGGGCCGCAG-3′; antisense, 5′-CTGCGGCCCAGGCCGCTGCTGC-3′
CMV F	Forward, 5′-CGCAAATGGGCGGTAGGCGTG-3′
EGFP R	Forward, 5′-CGTCGCCGTCCAGCTCGACCAG-3′
LMNB2	Forward, 5′-AGTTCACGCCCAAGTACATC-3′; reverse, 5′-CTTCACAGTCCTCATGGCC-3′
45S	Forward, 5′-GAACGGTGGTGTGTCGTT-3′; reverse, 5′-GCGTCTCGTCTCGTCTCACT-3′
28S	Forward, 5′-AGAGGTAAACGGGTGGGGTC-3′; reverse, 5′-GGGGTCGGGAGGAACGG-3′
18S	Forward, 5′-GATGGTAGTCGCCGTGCC-3′; reverse, 5′-GCCTGCTGCCTTCCTTGG-3′
MALAT1	Forward, 5′-GACGGAGGTTGAGATGAAGC-3′; reverse, 5′-ATTCGGGGCTCTGTAGTCCT-3′

### Act D treatment.

Cells were treated with 0.05 μg/ml actinomycin D (Act D) in complete medium for 4 h at 37°C with 5% CO_2_ after which they were lysed to obtain RNA or protein. Equivalent volumes of dimethyl sulfoxide (DMSO) were used as vehicle controls. Similarly, cells were treated with Act D and fixed for immunofluorescence, or live cells were imaged using confocal microscopy.

### Immunofluorescence assay (IFA).

Adherent DLD-1 cells were washed twice in 1× phosphate-buffered saline (PBS) (pH 7.4), permeabilized for 5 min with CSK buffer [0.1 M NaCl, 0.3 M sucrose, 3 mM MgCl_2_, 10 mM piperazine-*N*,*N*′-bis(2-ethanesulfonic acid) (PIPES) (pH 7.4), 0.5% Triton X-100] on ice, fixed in 4% paraformaldehyde (PFA) (Sigma, P6148) for 10 min, and repermeabilized in 0.5% Triton X-100 for 10 min. Cells were blocked in 1% bovine serum albumin (BSA) (Sigma, A2153) for 30 min and incubated with primary antibody (diluted in 0.5% BSA) for 1.5 h and with secondary antibodies (diluted in 1× PBS plus 0.1% Triton X-100 [PBST]) for 1 h. Cells were washed thrice in 1× PBS in between antibody incubations. Cells were counterstained with DAPI (4′,6′-diamidino-2-phenylindole) and mounted in SlowFade gold antifade (Invitrogen, S36937). The antibodies used are listed in Table 2.

**TABLE 2:** Antibodies used in this study

Antibody	Purpose	Dilution or amt
Antinucleolin (ab13541)	IFA	1:300
Antinucleolin (ab22758)	IFA	1:500
	Western blotting	1:2,000
	IP	2 μg
Antinucleolin (ab50279)	Immuno-RNA-FISH	1:500
Anti-lamin A/C (Epitomics, 2966S)	IFA	1:600
	Western blotting	1:5,000
Anti-lamin A/C (ab40567)	IFA	1:50
	Western blotting	1:200
Anti-lamin B2 (ab8983)	IFA	1:400
	Western blotting	1:400
Antifibrillarin (ab5821)	IFA	1:500
Antinucleophosmin (ab37659)	Western blotting	1:1,000
	IP	2 μg
Anti-p84 (ab487)	IFA	1:500
Antinucleophosmin (ab10530)	Western blotting	1:500
Antiactin	Western blotting	1:400
Anti-GAPDH	Western blotting	1:5,000
Anti-rabbit antibody–Alexa Fluor 488	IFA	1:1,000
Anti-rabbit antibody–Alexa Fluor 568	IFA	1:1,000
Anti-mouse antibody–Alexa Fluor 488	IFA	1:1,000
Anti-mouse antibody–Alexa Fluor 568	IFA	1:1,000
Anti-mouse antibody–Alexa Fluor 633	IFA	1:1,000
Donkey anti-rabbit antibody–horseradish peroxidase (GE, NA9340V)	Western blotting	1:10,000
Sheep anti-mouse antibody–horseradish peroxidase (GE, NA9310V)	Western blotting	1:10,000
Normal rabbit IgG	IP	2 μg

### Western blotting and co-IP.

SDS-PAGE and immunoblotting were performed as per standard protocols. Lysates were prepared in radioimmunoprecipitation assay (RIPA) buffer containing 1× protease inhibitory cocktail (PIC) (Roche), and the protein concentration was estimated using a bicinchoninic acid (BCA) kit (Pierce, 23225). Proteins were resolved by 10% SDS-PAGE and transferred to Immobilon-P membranes (GE). Blots were blocked in 5% nonfat dry milk. The blots were incubated with primary antibodies for 3 h at room temperature or overnight at 4°C, followed by incubation with secondary antibodies for 1 h at room temperature. Immunoblots were developed using the chemiluminescent substrate ECL Prime (GE, 89168-782) and imaged with ImageQuant LAS4000. The antibodies used are listed in Table 2.

For coimmunoprecipitation (Co-IP) assays, ∼10^7^ cells (DLD-1) were lysed in co-IP lysis buffer (50 mM Tris [pH 7.4], 150 mM NaCl, 0.5% NP-40, 1× PIC) vortexed and incubated on ice for 15 min, and centrifuged at 12,000 rpm and 4°C for 10 min. The lysate was precleared by incubating with Dynabeads protein A (Invitrogen, 10002D) for 1 h. Two micrograms of specific antibody or normal rabbit IgG was incubated with lysates overnight at 4°C. Protein A beads, preblocked with 0.5% BSA, were incubated with the immunocomplex for 2 to 3 h. Beads were washed 6 times with co-IP lysis buffer (plus 0.5 mM phenylmethylsulfonyl fluoride [PMSF]) to minimize nonspecific binding. Bound protein was eluted from the beads by boiling in 2× Laemmli buffer for 15 min at 95°C.

### Nucleolar isolation and immunostaining.

Nucleolar isolation was performed as described previously (84). Briefly ∼10^7^ cells (DLD-1) were washed and scraped in ice-cold solution I (0.5 ml; 0.5 M sucrose with 3 mM magnesium chloride [MgCl_2_] and 1× PIC). Cells were sonicated 5 times at 50% amplitude, 10 s on and 10 s off, on ice (Sonics Vibracell), layered over solution II (0.7 ml, 1.0 M sucrose, 3 mM MgCl_2_), an centrifuged at 1,800 × *g* for 5 min at 4°C. The supernatant was removed carefully. The nucleolar pellet was resuspended in 1× PBS, spotted on glass slides, air dried, and fixed with 4% PFA for 20 min. Nucleoli were blocked in 1% BSA for 60 min at room temperature and incubated with primary antibody and secondary antibodies for 30 min each, followed by three extensive washes with 1× PBST. The primary and secondary antibodies used were diluted in 1% BSA in 1× PBST. The preparation was mounted in DAPI-antifade. For lamin B2 knockdown, nucleoli were isolated by pooling cells from 3 independent wells of a 6-well plate.

### Nuclear matrix preparation.

Nuclear matrix was prepared from DLD-1 cells as previously described (85). DLD-1 cells grown on coverslips were washed thrice in ice-cold cytoskeletal buffer (10 mM PIPES [pH 6.8], 10 mM KCl, 300 mM sucrose, 3 mM MgCl_2_, 1 mM EDTA, 0.05 mM PMSF, 1× PIC) and then incubated for 10 min in CSK buffer containing 0.5% Triton X-100 at 4°C. Cells were rinsed thrice in ice-cold RSB buffer (42.5 mM Tris-HCl [pH 8.3], 8.5 mM NaCl, 2.6 mM MgCl_2_, 0.05 mM PMSF, 1× PIC) and incubated for 10 min in RSB buffer containing 1% (vol/vol) Tween 20 and 0.5% (vol/vol) sodium deoxycholate at 4°C. Cells were rinsed twice in ice-cold digestion buffer (10 mM PIPES [pH 8.3], 50 mM NaCl, 300 mM sucrose, 3 mM MgCl_2_, 1 mM EGTA, 0.05 mM PMSF, 1× PIC) and then incubated for 30 min in digestion buffer containing 100 U/ml DNase I (Roche) at 30°C. Ammonium sulfate (1 M) was added to the cells to a final concentration of 0.25 M and incubated for 5 min to remove digested chromatin, followed by two washes in ice-cold digestion buffer. Cells were incubated in 2 M NaCl for 5 min at 4°C, washed twice in digestion buffer, fixed in 4% PFA, and immunostained.

### Microscopy.

Cells were imaged on Zeiss LSM710 and LSM780 confocal microscopes with 405-nm, 488-nm, and 561-nm laser lines using a 63× Plan-Apochromat 1.4-numerical-aperture (NA) oil immersion objective at 2.0 to 2.5× digital zoom. Scanning was performed sequentially (*x-y*, 512 pixels by 512 pixels [1 pixel ∼ 0.105 μm]), and z-stacks were collected at a step size of 0.34 μm and a pinhole size of ∼0.7 μm (1 arbitrary unit [AU]). The pixel depth was 8 bits, the line averaging was 2, and the scan speed was 10. For superresolution imaging of isolated nucleoli, a Zeiss LSM800 with an Airyscan detector was used. For immuno-RNA-FISH experiments, fixed cells were imaged using a Leica TCS Sp8 microscope (*x-y*, 512 pixels by 512 pixels; *z*, 0.34 μm; frame averaging, 2; scan frequency, 400 Hz).

### Imaging and scoring of nucleolar morphologies.

Images were captured based on several random fields using DAPI staining and the extent of lamin knockdown in each nucleus. Nucleolar morphology was scored by inspecting nucleolin staining. Nucleoli were visually inspected across a number of independent nuclei. Intact nucleoli were spherical and discrete, whereas disrupted nucleoli were irregular aggregates.

### Live imaging of cells and FRAP analysis.

A Zeiss LSM710 or LSM780 confocal microscope equipped with a heated stage at 37°C was used for all photobleaching experiments and fluorescence image acquisitions. For live imaging, cells were grown on a 22- by 22-mm^2^ coverslip glued onto a 35-mm petri dish coated with 100 μg/ml collagen (BD Biosciences; 354236); CO_2_-independent Leibovitz L-15 medium (Gibco; 21083-027) was used during microscopy. To visualize nucleolin aggregates, cells were treated with Act D and imaged. z-stacks (∼0.34 μm) of cells were acquired every 1.3 min.

For fluorescence recovery after photobleaching (FRAP) analysis, images were acquired using a 63× oil immersion objective, NA 1.4 at 2.5× digital zoom, at 2% laser power to avoid photobleaching. A 1- by 1-pixel square (1 pixel = 0.11 μm) region of interest (ROI) was used for bleaching nucleolin speckles. Ten images were acquired before photobleaching. Photobleaching was performed using 200 iterations of the 488-nm laser line at 100% power. Images were collected every 484 ms for a total duration of 25 s. Images were analyzed using the Zen 2011 FRAP analysis module, and relative fluorescence intensity (RFI) was calculated as (86) RFI = {[ROI1(*t*) − ROI3(*t*)]/[ROI2(*t*) − ROI3(*t*)]} × {[ROI2(*t* = 0) − ROI3(*t* = 0)]/[ROI1(*t* = 0) − ROI3(*t* = 0)]}, where ROI1 is the fluorescence intensity of the 1- by 1-pixel ROI that is bleached, ROI2 is the total nucleus fluorescence intensity, and ROI3 is the fluorescence intensity of a 1- by 1-pixel background region selected outside the nucleus. ROI1(*t*) denotes the postbleach fluorescence intensity at time *t*. ROI2(*t*) and ROI3(*t*) denote the same for the total nucleus and background, respectively. ROI1(*t* = 0) denotes the average prebleach fluorescence intensity. ROI2(*t* = 0) and ROI3(*t* = 0) denote the same for the whole nucleus and background, respectively. The double-normalized data were transformed on a scale of 0 to 1. Mobile fractions and *t*_1/2_ were calculated by fitting the normalized data (without transformation) with double-exponential fit using easyFRAP software (87).

### qRT-PCR analysis.

RNA was prepared by lysing cells in TRIzol (Applied Biosciences) and phenol-chloroform extraction. cDNA was synthesized using the ImProm II reverse transcriptase system (Promega A3800). Quantitative real-time PCR (qRT-PCR) was performed using SYBR green (SAF Labs). The primers used are listed in Table 1. Actin served as an internal control.

### RNA-FISH and 3D-immuno-RNA-FISH. (i) Fixation.

RNA-FISH was performed as described previously (88). All reagents for RNA-FISH were prepared in diethyl pyrocarbonate (DEPC)-treated water and supplemented with 2 mM vanadyl ribonucleoside complex (New England BioLabs). Briefly, cells were washed with 1× PBS, permeabilized with CSK buffer on ice for 5 min, fixed in 4% PFA for 10 min, and stored in 70% ethanol at −20°C until hybridization. For 3D-immuno-RNA-FISH, after fixation in 4% PFA, cells were permeabilized with 0.5% Triton X-100 in PBS, and immunostaining was performed as described earlier (88). Cells were postfixed in 4% PFA for 10 min and washed twice with 2× SSC (1× SSC is 0.15 M NaCl plus 0.015 M sodium citrate), followed by hybridization.

### (ii) FISH probe labeling.

RNA-FISH probes for the ∼12-kbp intergenic sequence (IGS) upstream of the rDNA start site was prepared by nick translation of the pUC9-rDNA vector (a gift from Brian McStay) with Spectrum Red-conjugated dUTP, using DNase I (5 mU/μl)-DNA polymerase I (50 mU/μl) (Roche) for 3 h at 15°C. Six micrograms of nick-translated probe was precipitated overnight at −20°C with 20 μg human Cot1 DNA (Invitrogen) and 40 μg salmon sperm DNA (Invitrogen) using cold 100% ethanol and 1/10 volume of 3 M sodium acetate. The probe was resuspended in deionized formamide at 37°C. Prior to hybridization, the probe was denatured at 80°C for 5 min, followed by addition of an equal volume of 2× hybridization mix containing 2 mM vanadyl ribonucleoside and incubation on ice for 30 min.

### (iii) Hybridization and washes.

Cells for RNA-FISH were dehydrated in an ethanol series (70%, 90%, and 100% ethanol) and air dried. Approximately 1 μg of probe (∼4 to 6 μl) was spotted on an RNase-free slide, and cells on coverslips were inverted on the probe and sealed using nail varnish. Hybridization was carried out for 16 h at 37°C. Coverslips were washed thrice in 50% formamide–2× SSC (pH 7.2), followed by three washes in 2× SSC (pH 7.2), for 5 min each at 42°C and mounted in DAPI-antifade.

### Image processing and analysis.

3D volume rendering and analysis of nuclei and nucleoli were performed using Image Pro Plus v7.1. Volume measurements of nucleolin speckles were performed using the ImageJ object counter 3D plugin. Colocalization analysis was performed using the JACOP plugin from ImageJ (89). 4D time-lapse images were analyzed using Imaris 8.0.0.

### Statistical analysis and graphs.

Statistical analyses were performed for each experiment as described in the figure legends, and graphs were plotted using GraphPad Prism 5 software; a *P* value of <0.05 was considered significant. We imaged and analyzed a minimum of 20 to 30 cells for each biological replicate in fixed preparations, while a minimum of 2 or 3 nuclei were tracked and analyzed during prolonged live imaging of nucleolin speckles. In the figures and legends, *N* is the number of independent biological replicates and *n* is as defined for each figure.

## Supplementary Material

Supplemental material
