# Supplementary material for: Lamin B2 Modulates Nucleolar Morphology, Dynamics, and Function
Source: Mol Cell Biol. 2017 Nov 28;37(24):e00274-17. doi: 10.1128/MCB.00274-17 (PMC5705821; doi:10.1128/MCB.00274-17)
Supplement: Supplemental material [file supp_37_24_e00274-17__index.html]

Supplemental material 

# Lamin B2 Modulates Nucleolar Morphology, Dynamics, and Function

## Supplemental material

- Supplemental file 1 -

  Movie S1 (Control cells imaged from 1 h after Act D treatment)

  MP4, 8.6M
- Supplemental file 2 -

  Movie S2 (Lamin B2-depleted cells imaged 1 h after Act D treatment)

  MP4, 1.5M
- Supplemental file 3 -

  Movie S3 (Lamin B2-depleted cells imaged 2.5 h after Act D treatment)

  MP4, 2.0M
